# Association of body mass index with peer aggression, reaction to peer aggression and physical activity in rural Chinese children

**DOI:** 10.3389/fpubh.2025.1595005

**Published:** 2025-05-30

**Authors:** Sixiang Tao, Hongzhao Wang, Yaming Song, Denise Koh

**Affiliations:** ^1^Faculty of Education, National University of Malaysia, Bangi, Selangor, Malaysia; ^2^Faculty of Physical Education, Huainan Normal University, Huainan, Anhui, China

**Keywords:** body mass index, peer aggression, reaction to peer aggression, physical activity, rural children

## Abstract

**Objectives:**

To investigate the relationship between body mass index (BMI) and peer aggression, reaction to peer aggression, and physical activity in rural Chinese children.

**Materials and methods:**

The Chinese version of the Peer Aggression and Reactive Behavior Questionnaire assessed children's peer aggression and reaction to peer aggression; the Chinese version of the Physical Activity Questionnaire for Children assessed physical activity in the last week. Study was conducted in four rural junior high schools in Fuyang City, Anhui Province, and 356 first-grade students were finally included. Participants were classified into six categories (from −2SD to +3SD) according to the WHO BMI criteria for children. The Kruskal–Wallis H-test and Mann–Whitney U-test were used for between-group comparisons. Spearman correlation analyses and restricted cubic spline analyses were used to assess the relationships between BMI and behavioral indicators. All data analyses were done using R Studio.

**Results:**

BMI was significantly and non-linearly associated with peer aggression, which increased significantly after BMI exceeded +1SD (+2SD: *r* = 0.366, *p* < 0.01; +3SD: *r* = 0.807, *p* < 0.001). Physical activity tended to decrease with increasing BMI and was significantly negatively correlated with peer aggression in the high BMI group (*r* = −0.972, *p* < 0.001). Reaction to peer aggression increased with BMI, but the association was insignificant.

**Conclusions:**

BMI in rural children was significantly associated with peer aggression and physical activity, and physical activity may play a moderating role between BMI and aggression. This finding provides new ideas for the intervention of aggression in rural children and highlights the importance of integrating weight management, physical activity, and behavioral interventions.

## Introduction

Childhood aggression and bullying behavior have become an important global public health concern and are particularly prominent in rural China. Research has shown that bullying and aggressive behavior among school-aged children in rural China have been recognized as a serious social problem (1). Specific data show that the prevalence of aggressive behavior among adolescents in rural China is as high as 24.3% (2), and rural children are more likely to be involved in bullying than urban children (3). This situation is even more serious in the left-behind children group, where the study found that 31.6% of left-behind children reported frequent bullying, a significantly higher proportion than rural children living with their parents (1). These worrying statistics highlight the urgency of addressing bullying and aggressive behaviors in rural China, especially given the significant impact these behaviors have on children's psychosocial adjustment and life satisfaction (4).

The impact of aggressive behavior on children's development is multifaceted and manifests itself in a two-way negative effect (5). For the perpetrator, such behavior not only leads to rejection and isolation from peers but also affects his or her long-term social adjustment; for the victim, he or she may display persistent emotional and behavioral problems at school and home (6). It has been found that children who display more proactive aggression tend to lack a sense of guilt for harming others, and that this form of aggression is closely associated with externalizing symptoms such as antisocial behavior, psychopathology and delinquency. In contrast, children with reactive aggression were more likely to have mood regulation disorders such as anxiety and anger, as well as being at risk of more strained peer relationships, lower social acceptance and higher social isolation (7).

There is a significant association between weight status and aggressive behavior in children. A new meta-analysis confirmed that children and adolescents who were overweight or obese exhibited more physical aggression than their normal weight peers, and this effect was more pronounced in the male group (8). Longitudinal studies have further validated the association between aggressive behavior and higher BMI (9). Meanwhile, overweight and obese children were more likely to be targets of bullying, with obese children and overweight children having a significantly higher risk of bullying than their normal weight peers (10). Notably, severely obese children faced not only verbal insults but also physical harm and social exclusion, with obese boys more likely to be both bullies and victims (11). In addition, it was found that obese and overweight children exhibited a significantly higher prevalence of externalizing problems, including aggression and delinquent behavior, than normal weight children (12).

Physical activity as a potential intervention has a positive effect on mitigating aggressive behavior among children. A Systematic review and meta-analysis showed that regular physical activity was effective in reducing overall aggression scores and hostility levels in participants with aggressive tendencies (13). Physical activity was found to exhibit a significant negative relationship with active aggression and to significantly weaken the association between active aggression and peer criminal behavior at higher levels of physical activity (14). Evidence-based research further confirms that students who regularly participate in physical activity exhibit lower levels of aggression compared to those who do not, an effect that is reflected in all three dimensions of aggression toward self, others, and property (15). These findings provide an important theoretical basis for developing physical activity-based intervention strategies for aggressive behavior.

In summary, aggression and bullying among rural Chinese children is a social problem that needs to be urgently addressed, and its incidence is significantly higher than that of urban children. It has been shown that there is a strong correlation between children's weight status and different forms of aggressive behavior (aggressive and reaction to aggression), and that overweight and obese children are not only more likely to display aggressive behavior, but also more likely to be victims of bullying (9, 10). And physical activity, as a potential protective factor, may play an important role in moderating aggressive behavior. However, systematic studies on the interaction between these three factors (aggressive behavior, weight status and physical activity) in rural Chinese children's populations are still insufficient.

Based on the above discussion, the following hypotheses were proposed in this study: (1) there are significant differences in the patterns of peer aggression, reaction to peer aggression among rural Chinese children with different weight statuses; (2) there is a significant association between physical activity and rural Chinese children's peer aggression and reaction to peer aggression; (3) there is an interaction between weight status and physical activity on rural Chinese children's peer aggression and reaction to peer aggression. To test these hypotheses, we conducted a cross-sectional study of rural Chinese children to investigate the performance characteristics and their interrelationships of peer aggression, reaction to peer aggression, and children's physical activity in children with different weight status, to provide a scientific basis for the prevention and intervention of aggressive behavior among rural Chinese children.

## Materials and methods

### Participants

This study was conducted in rural areas of Fuyang City, Anhui Province, using convenience sampling with a cross-sectional survey design. The participants were first-grade students from four rural junior high schools. This grade level was explicitly selected because the first year of middle school (mean age 12.48 ± 0.58 years) represents a critical period for the formation of social status and peer relationship reorganization, as well as a developmental stage when weight status and social behaviors may become significantly associated (16). The research team focused on students from a single grade to control for age-related variables and ensure sample homogeneity. A total of 400 questionnaires were distributed, with schoolteachers reading the questions aloud while students completed the questionnaires independently. The complete set of questionnaires (including the Peer Aggression and Reactive Behavior Questionnaire and Physical Activity Questionnaire for Children) took ~20–25 min to complete. Of the 395 questionnaires collected, 9 were excluded due to incomplete responses or duplicate selections, and 30 were excluded because students reported illness or other factors affecting physical activity results in the preceding week on the PAQ-C questionnaire. Therefore, the final sample size was 356 participants. This sample size exceeds the threshold of 300 participants recommended as 'good' for statistical analyses in behavioral research (17). Additionally, *post-hoc* power analysis using G^*^Power (18) with an effect size of 0.25, alpha level of 0.05, and six groups indicated that this sample size achieved a statistical power of 0.97, well above the conventional 0.80 threshold. The study was approved by the Research Ethics Committee of Huainan Normal University (approval number: 2024-047). Written informed consent was obtained from all participants and their parents or legal guardians before data collection.

## Measure

### Peer aggression and reaction to peer aggression

For peer aggression and reaction to peer aggression survey study use Peer Aggression and Reactive Behavior Questionnaire, which contains 17 entries divided into two scales, Peer Aggression scales, (PA) and Reaction to Peer Aggression Scale (RPA). Both scales are in the form of a 4-point Likert scale. The PA scale asks respondents “Usually, how many times have you...?” The PA scale asked respondents “How many times do you usually...?”, with answers ranging from 1 (“never”) to 4 (“every day”). The RPA scale presented a situation and asked “Usually, when a classmate... (describe behavior), do you...?” Answers ranged from 1 (“I never do this”) to 4 (“I do this all the time”). The peer aggression and reaction to peer aggression were measured using the Chinese version of the Aggression Scale, with both subscales demonstrating good internal consistency (Cronbach's α = 0.81 for PA and 0.87 for RPA) (19).

### Physical activity

Physical activity were assessed using the Chinese version of the Physical Activity Questionnaire for Children (PAQ-C), which measures moderate-to-vigorous physical activity over the past 7 days. According to the PAQ-C manual, the questionnaire was administered to 8–14-year-olds (~grades 4–8), which is consistent with the age and school environment characteristics of the first-year middle school students in this study (20). The PAQ-C demonstrates satisfactory reliability (Cronbach's α = 0.79, composite reliability ρ = 0.81, intraclass correlation coefficient α = 0.82) and validity, with confirmatory factor analysis confirming a single-factor structure (21).

### BMI

Body mass index (BMI) was calculated using height and weight data collected as part of the Chinese National Student Physical Fitness Standard (CNSPFS) surveillance program (22). This surveillance was conducted by school physical education teachers in November 2024 (one month prior to the questionnaire survey) following the standardized CNSPFS protocols. Therefore, children were not weighed and measured again on the day of the questionnaire administration; instead, the most recent CNSPFS surveillance data were used, as this time interval would not significantly impact the research results. According to CNSPFS standards, height was measured to the nearest 0.1 cm using wall-mounted stadiometers, and weight was measured to the nearest 0.1 kg using calibrated electronic scales, with participants wearing light clothing and no shoes. These data were provided by the physical education teachers responsible for CNSPFS testing at the participating schools, in compliance with formal research collaboration agreements and ethical approval requirements. BMI was calculated as weight in kilograms divided by height in meters squared (kg/m^2^). BMI values were converted to *z*-scores based on the World Health Organization (WHO) growth reference for school-aged children and adolescents, and participants were categorized into seven groups according to standard deviation (SD) intervals: −3SD, −2SD, −1SD, median, +1SD, +2SD, and +3SD. These classifications correspond to underweight, normal weight, overweight, and obesity status, allowing for a standardized assessment of weight status in the study population.

### Statistical analysis

As the variables did not obey a normal distribution, as verified by Shapiro–Wilk tests (23), quantitative variables were characterized using mean and standard deviation (*M* ± SD) and 95% confidence intervals (95% CI). Differences in Peer Aggression (PA), Reaction to peer Aggression (RPA), and Physical Activity in Children (PA-C) were compared between the different BMI classification groups using the Kruskal–Wallis H-test, and when the test results showed significant differences, two-by-two comparisons were made using the Mann–Whitney U-test, and Bonferroni's method was used to correct for multiple comparisons were corrected.

To explore the relationship between BMI and behavioral indicators, Spearman rank correlation analysis was used to examine the correlation between BMI and peer aggression, reaction to peer aggression, and physical activity. The effect of BMI threshold on behavioral indicators was further explored by Restricted Cubic Spline Analysis (RCSA), in which the samples were grouped according to the standard deviation (±1SD, ±2SD, ±3SD) using normal BMI as a reference to analyze the non-linear trends of behavioral indicators at different BMI levels.

All statistical analyses in this study were done using R Studio. Correlation analyses were performed using the ggplot2 package to plot correlation matrices, and behavioral change curves were analyzed and visualized using the splines package for restricted cubic spline analysis. The statistical significance level was set at *p* < 0.05, and significance levels were marked with an asterisk in the graphs (^*^p < 0.05, ^**^p < 0.01, ^***^p < 0.001).

## Results

A total of 356 rural Chinese children were included in the study, and the descriptive statistics of the sample are shown in Table 1. The 95% confidence intervals (CIs) were calculated based on the standard error of the mean, regardless of the underlying distribution of the variables. Prior to analysis, we examined the distribution of all variables using Shapiro–Wilk tests and visual inspection of histograms.

**Table 1:** Descriptive statistics (*n* = 356).

**Variable**	**M ±SD**	**95%CI**	**Chi-square**	**df**	***p***
Age (years)	12.48 ± 0.58	[12.42, 12.54]			
Height (cm)	160.21 ± 8.27	[159.34, 161.08]			
Weight (kg)	51.63 ± 10.50	[50.53, 52.73]			
BMI (kg/m^2^)	20.07 ± 3.55	[19.70, 20.44]			
Peer aggression	8.07 ± 2.46	[7.81, 8.33]	15.23	5	0.009
Reaction to peer aggression	22.34 ± 5.63	[21.75, 22.93]	6.78	5	0.237
Physical activity	2.26 ± 0.64	[2.19, 2.33]	14.96	5	0.011

BMI values were converted to age- and sex-specific z-scores using the WHO Growth Reference for aged 5–19. Based on these z-scores, participants were classified into six categories: −2SD (1.4%), −1SD (9.6%), median (60.4%), +1SD (19.7%), +2SD (8.1%), and +3SD (0.8%).

The study population had a mean age of 12.48 years (SD = 0.58, 95% CI: 12.42–12.54), mean height of 160.21 cm (SD = 8.27, 95% CI: 159.34–161.08), mean weight of 51.63 kg (SD = 10.50, 95% CI: 50.53–52.73), and mean body mass index (BMI) of 20.07 kg/m^2^ (SD = 3.55, 95% CI: 19.70–20.44).

Regarding behavioral indicators, the peer aggression score averaged 8.07 (SD = 2.46, 95% CI: 7.818.33), reaction to peer aggression averaged 22.34 (SD = 5.63, 95% CI: 21.75–22.93), and physical activity averaged 2.26 (SD = 0.64, 95% CI: 2.19–2.33). Although some variables did not follow normal distribution, we reported means and standard deviations to facilitate comparison with other studies, while using appropriate non-parametric methods for subsequent analyses.

Table 2 demonstrates the differences in scores for peer aggression, reaction to peer aggression and physical activity across BMI classifications. The Kruskal–Wallis H-test showed significant differences in peer aggression and physical activity across BMI classification groups (both *p* < 0.01), while the difference in reaction to peer aggression scores did not reach statistical significance across groups (*p* > 0.05).

**Table 2:** Comparison of peer aggression, reaction to peer aggression and physical activity scores under different BMI (*n* = 356).

**BMI category**	***n***	**Peer aggression (M ±SD)**	**Mann–Whitney**	**Reaction to peer aggression** **(M ±SD)**	**Mann–Whitney**	**Physical activity(M ±SD)**	**Mann–Whitney**
−2SD	5	7.60 ± 1.67^a^	–	19.80 ± 4.21	–	2.73 ± 1.08^a**^	–
−1SD	34	7.76 ± 2.03^a^	*U =* 78, *p =* 0.744	21.82 ± 5.37	*U =* 65, *p =* 0.395	2.09 ± 0.55^ab^	*U =* 49, *p =* 0.037^*^
Median	215	7.80 ± 2.44^a^	*U =* 492, *p =* 0.812	22.44 ± 5.82	*U =* 430, *p =* 0.382	2.30 ± 0.65^a**^	*U =* 425, *p =* 0.207
+1SD	70	8.31 ± 2.44^ab^	*U =* 142, *p =* 0.347	21.67 ± 4.96	*U =* 155, *p =* 0.482	2.30 ± 0.65^a**^	*U =* 140, *p =* 0.326
+2SD	29	9.48 ± 2.41^b**^	*U =* 39, *p =* 0.066	23.79 ± 6.14	*U =* 52, *p =* 0.172	2.09 ± 0.46^ab^	*U =* 42, *p =* 0.042^*^
+3SD	3	11.67 ± 3.51^b**^	*U =* 2, *p =* 0.036^*^	27.00 ± 2.65	*U =* 2.5, *p =* 0.055	1.52 ± 0.34^b**^	*U =* 1, *p =* 0.025^*^

Different superscript letters (a, b) in the same column indicate significant differences between groups based on Mann–Whitney U-test.

^*^p <0.05, ^**^p <0.01 (Bonferroni corrected).

Further two-by-two comparisons showed that the higher BMI +2SD group (9.48 ± 2.41) and the +3SD category (11.67 ± 3.51) were significantly higher than the other groups in terms of peer aggression (*p* < 0.01). Specifically, the peer aggression of children with BMI in the −1SD to +1SD categories was similar (7.60 ± 1.67, 7.76 ± 2.03, and 7.80 ± 2.44, respectively), with a slight elevation in the +1SD group (8.31 ± 2.44), and a trend of a significant increase to the +2SD and +3SD groups.

In terms of physical activity, there was an overall decreasing trend in scores with increasing BMI classification. The lowest BMI-2SD group had the highest physical activity (2.73 ± 1.08), which was significantly higher than the other groups (*p* < 0.01). Especially in the highest BMI +3SD category group, the physical activity decreased to the lowest (1.52 ± 0.34), and the difference was statistically significant (*p* < 0.01) compared to all other groups.

Reaction to peer aggression, although showing a higher score (27.00 ± 2.65) in the +3SD group, the difference between the groups did not reach statistical significance, which may be related to the uneven distribution of the sample size, especially in the extreme BMI groups (−2SD and +3SD categories).

**Figure 1:**
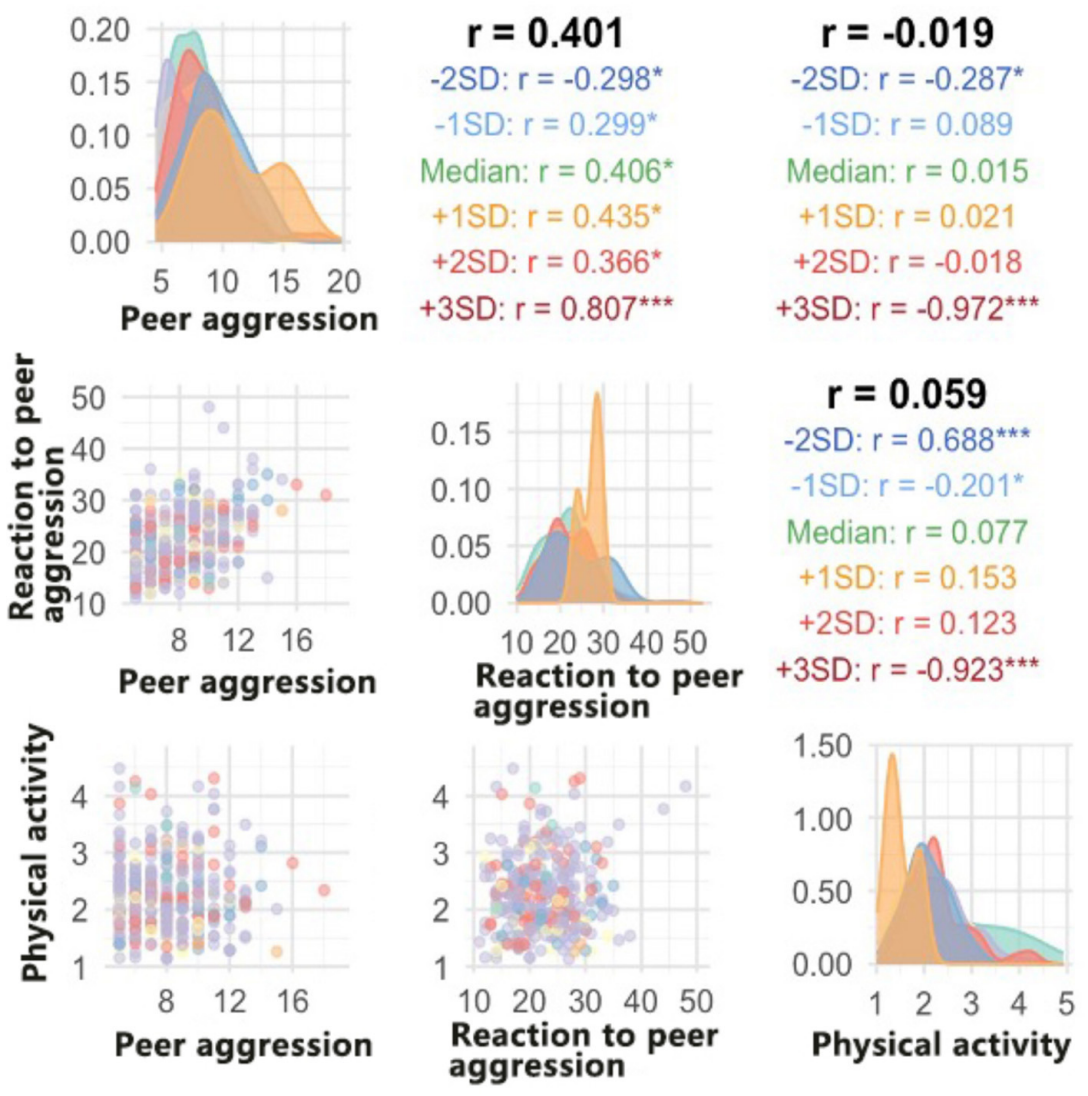
Correlation matrix and distribution patterns of peer aggression, reaction to peer aggression, and physical activity across different BMI categories.

Table 3 and Figure 1 illustrates the correlation matrix between the three behavioral indicators peer aggression, reaction to peer aggression, and physical activity. The results of the correlation analysis showed that. There was a moderate positive correlation between peer aggression and reaction to peer aggression (*r* = 0.401), and this correlation showed different strengths at different BMI levels. In particular, the correlation coefficient was significantly stronger in the higher BMI group (+3SD) (*r* = 0.807, *p* < 0.001), while a negative correlation was observed in the lower BMI group (−2SD) (*r* = −0.298, *p* < 0.05). This finding suggests that peer aggression and reaction to peer aggression may be simultaneously enhanced in children as BMI increases.

**Table 3:** Spearman correlation coefficients between BMI, peer aggression, reaction to peer aggression, and physical activity across different BMI categories.

**BMI category**	**Peer aggression vs. reaction to peer aggression**	**Peer aggression vs. physical activity**	**Reaction to peer aggression vs. physical activity**
Overall sample	*r =* 0.401^**^	*r =* −0.019	*r =* 0.059
−2SD	*r =* −0.298^*^	*r =* −0.287^*^	*r =* 0.688^***^
−1SD	*r =* 0.299^*^	*r =* 0.089	*r =* −0.201^*^
Median	*r =* 0.406^*^	*r =* 0.015	*r =* 0.077
+1SD	*r =* 0.435^*^	*r =* 0.021	*r =* 0.153
+2SD	*r =* 0.366^*^	*r =* −0.018	*r =* 0.123
+3SD	*r =* 0.807^***^	*r =* −0.972^***^	*r =* −0.923^***^

^*^p <0.05, ^**^p <0.01, ^***^p <0.001.

The overall correlation between peer aggression and physical activity was weak (*r* = −0.019) but showed significant differences across BMI levels. A strong negative correlation (*r* = −0.972, *p* < 0.001) was shown in the higher BMI group (+3SD), while a positive correlation (*r* = −0.287, *p* < 0.05) was shown in the lower BMI group (−2SD). This suggests that there may be a mutually inhibitory relationship between peer aggression and physical activity in overweight children.

The overall correlation between reaction to peer aggression and physical activity was also weak (*r* = 0.059) but showed significant correlations at the extremes of BMI. A strong negative correlation (*r* = −0.923, *p* < 0.001) was shown in the highest BMI group (+3SD), while a significant positive correlation (*r* = 0.688, *p* < 0.001) was shown in the lowest BMI group (−2SD). This result suggests that BMI may play a moderating role in the relationship between reaction to peer aggression and physical activity.

**Figure 2:**
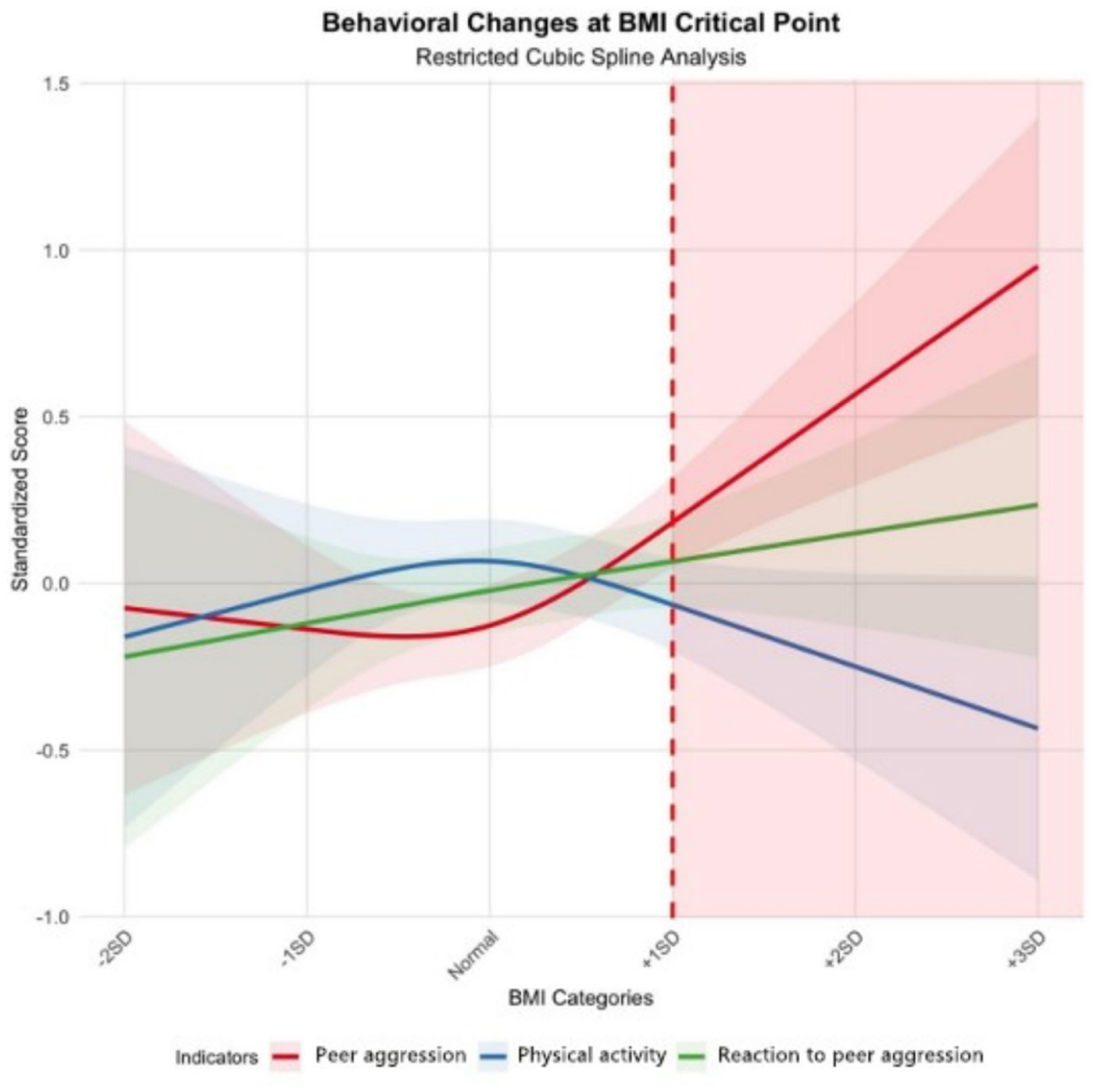
Behavioral changes at BMI critical point.

Figure 2 demonstrates the effect of the BMI threshold on the three behavioral indicators (peer aggression, reaction to peer aggression, physical activity) obtained by restricted cubic spline analysis. The analysis results show that these behavioral indicators show different trends before and after the BMI threshold (+1SD).

The peer aggression (red line) was relatively stable before the BMI threshold but showed a significant upward trend after exceeding the threshold. Specifically, peer aggression standardized scores began to increase significantly when BMI exceeded +1SD and showed the steepest upward trend in the +2SD to +3SD interval. This suggests that overweight and obese children may exhibit more peer aggressive behavior.

The reaction to peer aggression (green line) showed a relatively stable and slow upward trend overall, with relatively little change in its slope. Even after the BMI threshold, the increasing trend of RPA remained relatively flat, suggesting that the effect of BMI on reactive aggression may not be as significant as on proactive aggression.

The physical activity (blue line), on the other hand, showed the opposite pattern of change from peer aggression. Physical activity scores were relatively stable until the BMI threshold but began to decline significantly after the threshold was exceeded. This downward trend was most pronounced in the higher BMI range (+2SD to +3SD), suggesting that overweight and obese children may have significantly lower levels of physical activity than normal weight children.

The confidence intervals (shaded) of these curves show that the uncertainty in the predictions is greater at the extremes of BMI (especially the ±3SD interval), which may be related to the smaller sample sizes in these intervals.

The standardized scores at different BMI categories derived from the restricted cubic spline analysis are presented in Table 4, providing numerical values corresponding to the trends shown in Figure 2. As indicated in both the figure and table, the critical point at +1SD BMI marks the significant change in behavioral patterns, particularly for peer aggression which increases substantially beyond this threshold.

**Table 4:** Standardized scores of behavioral indicators at different BMI categories from restricted cubic spline analysis.

**BMI category**	**Peer aggression**	**Physical activity**	**Reaction to peer aggression**
−2SD	−0.1	−0.15	−0.2
−1SD	−0.15	0.2	−0.1
Median	−0.12	0.1	0
+1SD (critical point)	0.25	0	0.1
+2SD	0.6	−0.25	0.2
+3SD	0.95	−0.45	0.25

Values represent standardized scores derived from restricted cubic spline analysis.

## Discussion

### Association between BMI and peer aggression: non-linear relationship

Firstly, the present study found a significant non-linear association between BMI and peer aggression, especially after BMI exceeded the normal range, with a significant upward trend in active aggression (+2SD: *r* = 0.366, *p* < 0.01; +3SD: *r* = 0.807, *p* < 0.001). This finding not only validates previous studies in which overweight or obese children showed more physically aggressive behaviors (8), but further reveals a significant enhancement of this association after the BMI threshold. This non-linear association aligns with developmental systems theory (24), which emphasizes how physical characteristics can interact with social contexts to shape behavioral outcomes. The significant increase in aggression after the BMI threshold may reflect both biological mechanisms related to hormonal changes associated with higher body fat (25) and psychosocial processes wherein adolescents use aggression as a compensatory mechanism to establish social dominance when facing weight-related stigma (26). Unlike earlier studies that found primarily linear relationships between weight status and aggression our finding of a threshold effect suggests critical developmental periods when interventions might be most effective. This phenomenon may be related to the fact that overweight and obese children are more likely to play the dual role of bully and victim simultaneously (11), and this dual role may have exacerbated their aggressive performance.

### BMI, physical activity, and aggression: moderating relationships

Second, the present study observed a significant decreasing trend in physical activity with increasing BMI and a significant negative correlation between physical activity and peer aggression in the higher BMI group (*r* = −0.972, *p* < 0.001). This finding supports the conclusions of previous studies regarding the positive moderating effect of physical activity on aggressive behavior (13, 14). Notably, this association was more pronounced in overweight and obese children, which may indicate greater intervention value of physical activity in the high BMI child population. The finding in previous studies that children involved in organized physical activity had a lower risk of obesity and that sedentary behavior was associated with an increased risk of childhood obesity further supports the promotion of physical activity as an intervention strategy to reduce childhood obesity and related behavioral problems (27).

The moderating effect of physical activity on the BMI-aggressiveness relationship may act through multiple pathways. From a physiological perspective, regular physical activity may improve metabolic health and reduce levels of inflammation associated with obesity (28). From a psychological perspective, participation in physical activity promotes self-control and emotion regulation, and these enhanced abilities may reduce the likelihood that children will exhibit aggressive behavior (29). These mechanisms may be particularly important in rural Chinese children, who often face multiple psychosocial stressors.

Third, although reaction to peer aggression rose with increasing BMI, this association was less significant than active aggression, a finding that complements previous research on externalizing problems in obese and overweight children (12). Such differential results suggest that different types of aggression and response to aggression may be modulated by different mechanisms of influence, which has important implications for the development of targeted intervention strategies. Particularly in the specific group of rural Chinese children (1, 2), there is a need to integrate weight management, physical activity promotion and peer aggression interventions into systematic health promotion programs.

### Implications for rural Chinese children

The findings of this study are of relevance to rural Chinese children, especially left-behind children. Previous studies have documented a higher prevalence of obesity and behavioral problems in left-behind children (1), which could form a potential risk combination given our findings on the relationship between BMI and aggressive behavior. It has been suggested that rural Chinese children may face more environmental constraints related to physical activity compared to urban Chinese children (30), such as a lack of suitable sports facilities and venues, which may exacerbate the association between weight and behavioral problems. The dramatic increase in aggression after the BMI threshold suggests that weight management programs in rural schools should be designed for children close to or exceeding a +1SD BMI threshold that incorporate specialized components for children near or above the +1 SD BMI threshold.

### Limitations and future research directions

Despite the importance of the study's findings, several limitations should be noted. The cross-sectional design prevented causal inferences about the relationship between BMI, aggression, and physical activity. Longitudinal studies tracking these variables at multiple time points would provide stronger evidence of developmental trajectories and causal pathways (31). Additionally, while our BMI categorization followed WHO standards, future research could consider incorporating more direct measures of body composition. Future research should also explore potential gender differences in these relationships, as previous studies have found different patterns of associations between BMI and aggressive behavior between boys and girls (11).

## Conclusions

The study showed that there was a significant association between BMI and peer aggression, reaction to peer aggression and physical activity in rural Chinese children. It was found that peer aggression significantly increased and physical activity significantly decreased after BMI exceeded the threshold, and these two indicators showed a significant negative correlation in the high BMI group. At the same time, physical activity may play a moderating role between BMI and aggression, and this moderating effect was particularly significant in overweight and obese children. These findings provide new perspectives on the prevention and intervention of aggression in rural children and highlight the need for integrated interventions that focus on both physical activity and aggression in weight management.

## Data Availability

The raw data supporting the conclusions of this article will be made available by the authors, without undue reservation.
